# Effects of soybean meal concentration in lactating sow diets on sow and litter performance and blood criteria^1^

**DOI:** 10.1093/tas/txaa037

**Published:** 2020-03-31

**Authors:** Kiah M Gourley, Jason C Woodworth, Joel M DeRouchey, Mike D Tokach, Steve S Dritz, Robert D Goodband

**Affiliations:** 1 Department of Animal Sciences and Industry, College of Agriculture, Kansas State University, Manhattan, KS; 2 Department of Diagnostic Medicine/Pathobiology, College of Veterinary Medicine, Kansas State University, Manhattan, KS

**Keywords:** crude protein, lactation, litter performance, sow, soybean meal

## Abstract

A total of 131 sows (Line 241; DNA, Columbus, NE) were used in a study to evaluate the effect of increasing soybean meal concentration in lactating sow diets on sow and litter performance. Sows were blocked by body weight (BW) within parity on day 112 of gestation and allotted to one of three treatments of increasing dietary soybean meal (25%, 30%, or 35% of the total diet). Diets were formulated to 1.05% standardized ileal digestible lysine (Lys) with L-Lys HCl decreasing as soybean meal increased. All other amino acids and nutrients were formulated to meet nutrient requirement recommendations. Diets were fed from day 112 of gestation until weaning (day 20 ± 2). Litters were cross-fostered up to 48 h after farrowing to equalize litter size. Increasing soybean meal concentration increased (linear, *P* = 0.017) sow BW loss and tended to increase (quadratic, *P* = 0.052) sow backfat loss from farrowing to weaning. Sow average daily feed intake (ADFI) from day 0 to 7 was similar (*P* > 0.10) across dietary treatments. However, from day 7 to 14, 14 to weaning, and overall, ADFI decreased (linear, *P* = 0.01) as soybean meal concentration increased. Despite the linear response in ADFI, the greatest decrease was observed as soybean meal concentration increased from 30% to 35% of the diet. There was no evidence for difference (*P* > 0.10) in wean-to-estrus interval, litter size, litter weight, or litter weight gain between dietary treatments. Sow serum urea nitrogen concentrations taken on day 14 of lactation increased (linear, *P* = 0.001) as soybean meal concentration increased. There was no difference (*P* > 0.05) for sow creatinine concentration, regardless of dietary treatment, suggesting that the increased urea nitrogen was a reflection of the increased dietary crude protein as opposed to increased protein catabolism. In summary, sow feed intake decreased and weight loss increased as soybean meal concentration of the diet increased, with the greatest decrease observed at 35% of the total diet. Although there were no differences in litter performance, it appeared that 35% soybean meal in the lactation diet negatively affected feed intake.

## INTRODUCTION

Encouraging sow feed intake during lactation is one of the most critical factors in achieving maximum productivity in the farrowing house. Increased feed intake is associated with improved litter performance and sow reproductive performance (Koketsu et al., 1996). It is important that diet ingredient composition does not negatively affect lactation feed intake. A previous study (Yang et al., 2000a) observed a decrease in lactation average daily feed intake (ADFI) as total lysine (Lys) increased from 0.60% to 1.60%. While the researchers hypothesized that the decrease in intake was due to elevated serum urea nitrogen levels and varying branch chain amino acid ratios across their experimental diets, the soybean meal concentration also increased from 12.6% to 48.5% of the diet. A more recent study (Gourley et al., 2017) observed a decrease in feed intake when soybean meal increased above 29% of the total diet as total Lys concentration was increased.

To meet the standardized ileal digestible (SID) Lys requirement of the high-producing sow, both soybean meal and crystalline Lys are typically added to the diet; however, the question remains whether a maximum dietary concentration of soybean meal should be considered? To our knowledge, there is no previous research that has evaluated this question, while keeping Lys constant in dietary treatments. Therefore, the objective of the current study was to determine if the soybean meal level in lactation diets affects sow performance and feed intake.

## MATERIALS AND METHODS

The Kansas State University Institutional Animal Care and Use Committee approved the protocol used in this experiment. The experiment was conducted at the Kansas State University Swine Teaching and Research Center (Manhattan, KS).

A total of 131 sows (Line 241; DNA, Columbus, NE) and litters (241 × 600, DNA Genetics, Columbus, NE) were used across five batch farrowing groups (February to April and July to September 2018). Sows were individually housed in an environmentally controlled and mechanically ventilated barn. Each farrowing crate was equipped with a nipple waterer and electronic feeding system (Gestal Solo Feeder, Jyga Technologies, St-Lambert-de-Lauzon, Quebec, Canada). On day 112 of gestation, sows were weighed using a scale and moved into the farrowing house. Females were blocked by initial body weight (BW) within parity and allotted to one of the three dietary treatments within the farrowing group. Dietary treatments were corn-soybean meal based and consisted of three concentrations of soybean meal (25%, 30%, or 35% of the diet; Table 1). L-Lys HCl was decreased in the diets as soybean meal increased in order to formulate all diets to 1.05% SID Lys. Other feed-grade amino acids (Met, Thr, Trp, and Val) were added as needed to maintain a similar ratio to Lys. All other nutrients met or exceeded the NRC (2012) requirement estimates. Gestation diets fed prior to the study contained 0.56% SID Lys and 15% soybean meal. Sows received 2 kg/d of the gestation diet until entry into the farrowing house (day 112).

**Table 1.** Diet composition (as-fed basis)^*a*^

	Soybean meal, %		
Ingredient, %	25	30	35
Corn	67.97	63.38	58.84
Soybean meal, 46.5% CP	25.00	30.00	35.00
Choice white grease	2.00	2.00	2.00
Limestone	1.28	1.25	1.23
Monocalcium phosphate, 21%	1.80	1.78	1.73
Sodium chloride	0.50	0.50	0.50
L-Lys-HCl	0.34	0.18	0.03
DL-Met	0.09	0.05	0.00
L-Thr	0.17	0.10	0.03
L-Trp	0.03	0.00	0.00
L-Val	0.20	0.12	0.03
Trace mineral premix^*b*^	0.15	0.15	0.15
Vitamin premix^*c*^	0.50	0.50	0.50
Total	100	100	100
Calculated analysis			
SID amino acids, %			
Lysine	1.05	1.05	1.05
Isoleucine:lysine	60	68	76
Leucine:lysine	130	141	153
Methionine:lysine	32	30	28
Methionine and cysteine:lysine	56	56	56
Threonine:lysine	67	67	67
Tryptophan:lysine	20	20	23
Valine:lysine	85	85	85
TBCAA^*d*^:lysine	275	295	314
Valine:leucine	65	60	55
Isoleucine:leucine	46	48	50
Total lysine, %	1.18	1.19	1.20
Metabolizable energy, kcal/kg	3,331	3,322	3,316
Net energy, kcal/kg	2,511	2,478	2,447
SID lysine:NE, g/Mcal	4.25	4.31	4.37
CP, %	18	20	22
Ca, %	0.89	0.89	0.89
P, %	0.74	0.76	0.77
STTD P, %	0.50	0.50	0.50

STTD, standardized total tract digestible; TBCAA, Total branched chain amino acids.

^*a*^Sows were fed 2.7 kg/d from day 112 of gestation until farrowing, then ad libitum from farrowing until weaning.

^*b*^Provided per kilogram of diet: 121 mg Zn from zinc sulfate; 121 mg Fe from iron sulfate; 36 mg Mn from manganese oxide; 18 mg Cu from copper sulfate; 0.3 mg I from calcium iodate; 0.3 mg Se from sodium selenite; and 0.12 mg chromium picolinate.

^*c*^Provided per kilogram of diet: 8,818 IU vitamin A; 2,204 IU vitamin D; 66 IU vitamin E; 4.4 mg vitamin K; 0.04 mg vitamin B12; 83 mg niacin; 28 mg pantothenic acid; 8.3 mg riboflavin; 0.22 mg biotin; 1.65 mg folic acid; 2.2 mg pyridoxine; 551 mg choline; and 50 mg carnitine.

^*d*^Calculated TBCAA:Lys = Ile:Lys + Leu:Lys + Val:Lys.

Diets were manufactured at the Kansas State University O.H. Kruse Feed Mill in Manhattan, KS. A new batch of each treatment diet was manufactured for each farrowing group and packaged in 22.7-kg bags. During bagging, feed samples were collected from every fifth bag, pooled, and used for nutrient analysis.

From day 112 of gestation until farrowing (approximately day 115), sows were fed 2.47 kg/d of their respective treatment diets. Postpartum, sows were allowed ad libitum access to feed. Feed was weighed and added to a bin in front of each farrowing crate and used to feed each respective sow. Feed intake was recorded by weighing the amount of feed placed in the feeder and the amount remaining in the bin every 7 d until weaning. Sow BW and backfat depth (measured at the P2 position; Renco Lean Meter, S.E.C. Repro Inc., Quebec, Canada) were recorded at 24 h after farrowing and at weaning (day 20 ± 2). Within 48 h postpartum, piglets were processed and cross-fostered, regardless of dietary treatment, in an attempt to equalize litter size (minimum of 12 pigs per litter). Litters were weighed on days 2, 7, and 14 and at weaning. Litter average daily gain (ADG) was calculated as: (litter weaning weight – day 2 litter weight)/(days from day 2 to weaning). Preweaning mortality was calculated as the number of pigs weaned per sow divided by the number of pigs on day 2.

On day 14 of lactation, sows were fasted for 10 h and 10 mL of blood was collected via jugular venipuncture. Blood samples were centrifuged and serum was collected and then stored at −80 °C until analysis. At weaning, sows were moved to a breeding barn, individually housed, and checked daily for signs of estrus using a boar. The wean-to-estrus interval (WEI) was determined as the number of days between weaning and when sows were first observed to show a positive response to the back-pressure test.

Calculations for maternal empty body weight (EBW), body lipid (BL), and body protein (BP) at farrowing and weaning were made using Eq. 8–49, 8–50, and 8–51 from NRC (2012) as follows:

$$\begin{array}{l} \begin{array}{ll} & Maternal\;EBW\left(kg\right)=0.96\times maternalBW\\ & Maternal\;BL\left(kg\right)=-26.4+0.221\times maternalEBW\\ & \;+1.331\times P2backfat\\ & Maternal\;BP\left(kg\right)=2.28+0.178\times maternalEBW\\ & \;-0.333\times P2backfat \end{array} \end{array}$$

where backfat is measured in millimeter and BW is in kilogram.

### Chemical Analysis

Five samples (one pooled sample per farrowing batch) per dietary treatment were sent to a commercial laboratory and analyzed in duplicate (Ward Laboratories, Kearney, NE) for crude protein (CP; method 990.03; AOAC, 2006), Ca, and P (method 985.01; AOAC, 1990). Serum samples were analyzed in duplicate for serum urea nitrogen (Urea Nitrogen Colorimetric Detection Kit; Arbor Assays; Ann Arbor, MI) and creatinine (Creatinine Colorimetric Assay Kit; Cayman Chemical; Ann Arbor, MI).

### Statistical Analysis

Data were analyzed using generalized linear mixed models where dietary treatment was a fixed effect, with the random effects of farrowing group and block. Statistical models were fitted using the GLIMMIX procedure of SAS (Version 9.4, SAS Institute, Inc. Cary, NC). Preplanned linear and quadratic contrast statements were used to evaluate increasing soybean meal concentrations.

Sow ADFI, BW, backfat depth, litter weight, litter gain, lactation length, maternal empty BW, maternal BL and BP, serum urea nitrogen, and creatinine were evaluated assuming a normal distribution of the response variable. Litter weight on day 2 was used as a covariate for litter weights on days 7 and 14, and weaning litter weights, and litter weight gain to improve the fit of the model. In these cases, assumptions for normal distribution were checked using standardized residuals.

Litter counts and the WEI were fit using a negative binomial distribution. Piglet survivability was fit using a binomial distribution. Statistical models were implemented using the GLIMMIX procedure of SAS (Version 9.4, SAS Institute, Inc., Cary, NC). All results were considered significant at *P* ≤ 0.05 and marginally significant at 0.05 ≤ *P* ≤ 0.10.

## RESULTS

**Figure 1.**
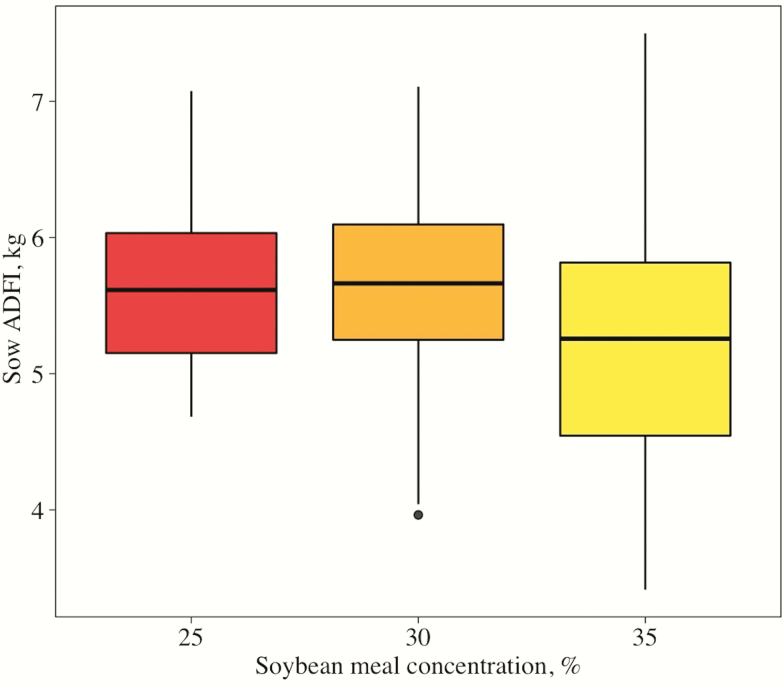
Box plot of overall sow ADFI by soybean meal level. The horizontal line in each box denotes the treatment median feed intake, while vertical lines indicate variation.

Chemical analysis of CP, Ca, and P were similar to formulated values (Table 2). There was no evidence for difference (*P* < 0.10) among treatments in initial BW or backfat depth measured after farrowing (Table 3), which validates the randomization of treatments. Increasing soybean meal concentration increased (linear, *P* = 0.017) sow BW loss and tended to increase (quadratic, *P* = 0.052) sow backfat loss from farrowing to weaning. Sow ADFI from day 0 to 7 was similar (*P* > 0.05) across treatments. However, from day 7 to 14, day 14 to weaning, and overall, ADFI decreased (linear, *P* < 0.001) as dietary soybean meal concentration increased. Additionally, overall ADFI appeared to be more variable as soybean meal concentration increased in the diet (Figure 1).

**Table 2.** Chemical analysis of experimental diets (as-fed basis)^*a*^

	Soybean meal, %				
Item, %	25	30	35
Dry matter	88.6	88.9	89.9
CP	18.3	20.1	22.1
Ca	1.03	1.04	1.08
P	0.71	0.72	0.78

^*a*^Diet samples were collected from each batch of feed at manufacturing from every fifth bag. Nutrient analysis was conducted in duplicate on composite samples (Ward Laboratories, Kearney, NE). Thus, each sample is a mean of 10 observations.

**Table 3.** Effects of increasing soybean meal concentration fed during lactation on sow performance^*a*^

	Soybean meal, %			Probability, *P *		
	25	30	35	SEM	Linear	Quadratic
Number of sows, *n*	44	43	44	–	–	–
Parity	2.0	2.0	2.0	0.15	0.998	0.157
Sow BW, kg						
Farrow	219.7	219.0	220.9	4.00	0.619	0.537
Wean	212.5	211.9	209.9	3.84	0.363	0.768
Change (farrow to wean)	−7.3	−7.0	−11.1	1.26	0.017	0.110
Sow backfat, mm						
Farrow	15.9	15.4	16.1	0.51	0.702	0.195
Wean	13.7	13.5	13.3	0.41	0.379	0.985
Change (farrow to wean)	−2.3	−1.9	−2.8	0.31	0.100	0.052
Sow ADFI, kg						
Days 0–7	3.6	3.6	3.6	0.11	0.684	0.798
Days 7–14	6.5	6.4	6.0	0.13	0.001	0.234
Day 14 to wean	7.3	7.1	6.5	0.16	0.001	0.227
Farrow to wean	5.7	5.6	5.2	0.11	0.001	0.314
Lactation length, d	19.5	19.5	19.3	0.14	0.319	0.299
Wean to estrus, d	4.5	4.4	4.4	0.12	0.618	0.891
Serum concentration^*b*^, mg/dL						
Urea nitrogen	20.4	25.4	28.1	1.14	0.001	0.318
Creatinine	3.7	3.6	3.8	0.19	0.580	0.584

^*a*^A total of 131 sows (Line 241; DNA, Columbus, NE) and their litters were used in a 21-d study.

^*b*^On day 14 of lactation, sows were fasted for 10 h and then bled. Samples were centrifuged after collection, and serum was used in analysis.

Calculated sow maternal EBW, BP, and BL were similar (*P* < 0.10) at farrowing (Table 4). Sow maternal empty BW loss increased (linear, *P* = 0.160) as soybean meal concentration increased. Sow maternal BL loss increased (quadratic, *P* = 0.028), where sows fed 35% soybean meal diets had greater BL mobilization compared with sows fed diets with 25% or 30% soybean meal. Maternal BP loss marginally increased (linear, *P* = 0.090) as dietary soybean meal concentration increased.

**Table 4.** Effects of increasing soybean meal concentration fed during lactation on sow body composition^*a*^

	Soybean meal, %				Probability, *P*	
	25	30	35	SEM	Linear	Quadratic
Number of sows, *n*	44	43	44	–	–	–
Maternal empty BW^*b*^, kg						
Farrow	210.9	210.2	212.0	3.83	0.619	0.537
Wean	204.0	203.4	201.5	3.66	0.362	0.768
Change, farrow to wean	−6.7	−6.4	−10.7	1.19	0.016	0.110
Maternal BL^*c*^, kg						
Farrow	41.3	40.6	41.8	1.19	0.618	0.261
Wean	36.8	36.4	35.7	1.09	0.311	0.817
Change, farrow to wean	−4.47	−3.97	−6.19	0.53	0.016	0.028
Maternal BP^*d*^, kg						
Farrow	34.5	34.5	34.7	0.66	0.711	0.876
Wean	34.1	34.0	33.7	0.62	0.455	0.785
Change, farrow to wean	−0.45	−0.51	−0.93	0.20	0.090	0.467

^*a*^A total of 131 sows (Line 241; DNA, Columbus, NE) and their litters were used in a 21-d study.

^*b*^Maternal EBW (kg) = 0.96 × maternal BW (Eq. 8–49; NRC, 2012).

^*c*^Maternal BL (kg) = −26.4 + 0.221 × maternal EBW + 1.331 × P2 backfat (Eq. 8–50; NRC, 2012).

^*d*^Maternal BP (kg) = 2.28 + 0.178 × maternal EBW − 0.333 × P2 backfat (Eq. 8–51; NRC, 2012).

There was no evidence for litter count at day 2 or weaning to be different (*P* > 0.10; Table 5) and, as a result, no evidence for differences in piglet survivability was observed (*P* > 0.10) across dietary treatments. There was no evidence for difference (*P* > 0.10) in litter weight on days 2, 7, and 14 or at weaning, or litter ADG, regardless of dietary treatment.

**Table 5.** Effects of increasing soybean meal concentration fed during lactation on litter performance^*a*^

	Soybean meal, %			Probability, *P *		
	25	30	35	SEM	Linear	Quadratic
Number of sows	44	43	44	–	–	–
Litter count, *n*						
Day 2^*b*^	13.7	13.6	13.6	0.56	0.863	0.988
Wean	13.0	12.9	12.9	0.04	0.963	0.955
Piglet survivability,^*c*^ %	95.2	95.0	95.7	1.00	0.654	0.651
Litter weight, kg						
Day 2	20.5	20.2	19.8	0.51	0.297	0.886
Day 7^*d*^	33.0	32.4	33.0	0.35	0.867	0.136
Day 14^*d*^	55.1	54.0	55.5	0.75	0.697	0.128
Wean^*d*^	70.2	69.2	70.2	1.29	0.995	0.414
Litter ADG, g^*d*^	3,002	2,937	3,032	72.7	0.724	0.288

^*a*^A total of 131 sows (Line 241; DNA, Columbus, NE) and their litters were used in a 21-d study.

^*b*^Cross-fostering occurred irrespective of treatment in an attempt to equalize litter size. Litters were weighed at 48 h after cross-fostering.

^*c*^Piglet survivability = litter count at weaning per litter count on day 2.

^*d*^Litter weight on day 2 was used as a covariate to improve the fit of the model.

There was no evidence for a difference in lactation length or WEI (*P* > 0.10) across dietary treatments. Sow serum urea nitrogen concentrations increased (linear, *P* < 0.001) as soybean meal concentration increased; however, there was no evidence for difference (*P* > 0.10) in creatinine concentration.

## DISCUSSION

In the present study, linear increases in sow BW, backfat, and maternal lipid loss were observed with increasing soybean meal concentration and was most evident with sows fed 35% soybean meal. This is likely a result of decreased lactation feed intake when sows were consuming the 35% soybean meal diet compared with 25–30% soybean meal diets. Decreased feed intake has also been observed in Lys titration studies where increasing Lys concentration by increasing soybean meal concentration from 14.5% to 48.5% (Yang et al., 2000a) or from 19% to 34% (Gourley et al., 2017) resulted in decreased sow feed intake. In contrast, Greiner et al. (2018) observed no change in feed intake when soybean meal concentration increased from 24.6% to 34% of the diet, while balancing diets to 1.12% SID Lys. In their study, however, feed intake was limited to a preset amount based on parity, which may have limited the ability to find a detectable difference as compared with ad libitum access to the feed intake used in our study. Touchette et al. (1998) conducted a Lys titration study during lactation with increasing soybean meal concentration from 18% to 43% and observed no change in feed intake in primiparous sows; however, feed intake was much lower in their study (3.9–4.1 kg/d) compared with ours, which also may have limited the ability to find a detectable difference.

It is well documented that when lactation feed intake is inadequate to support litter growth, the sow will mobilize body tissue to compensate in an attempt to maintain litter growth (Eissen et al., 2003; Yang et al., 2009; Ocepek et al., 2016). Typically, BL stores will be mobilized to meet energy deficiency during lactation before protein mobilization (Dourmad et al., 2008). This was validated in the current study with greater maternal BL mobilization compared with BP when voluntary feed intake was reduced and BW loss occurred.

The differences in feed intake response to increasing soybean meal could be due to an imbalance of amino acids in the diet or antinutritional factors in soybean meal. Branched-chain amino acids (BCAA) Val, Leu, and Ile are known to compete for the same AA transporters in the blood-brain barrier as Trp, a precursor for serotonin (Fernstrom, 2005). When the BCAA content of the diet increases, brain BCAA concentrations increase, while large neutral AA decrease, resulting in decreased neurotransmitter synthesis (Fernstrom, 2005). Furthermore, BCAA also share the same first step in catabolism, and excess of one BCAA, especially Leu, may expedite the degradation of others, resulting in decreased circulatory levels of Val and Ile (Brosnan and Brosnan, 2006). Total BCAA:Lys increased in the current study from 275% to 314% as soybean meal concentration increased. Recently, Millet et al. (2015) observed that growing pigs fed increasing levels of Leu had decreased growth and feed intake; however, this was able to be partially recovered by increasing the Val:Leu ratio. Similarly, a Val titration study with weaned pigs observed more severe feed intake reductions when Val:Leu ratios decreased (Meyer et al., 2017).

Data in lactating sows is more limited when evaluating relationships between BCAA. Previously, BCAA ratios had been investigated in lactating sows, and no interactions between BCAA were observed for feed intake or litter performance (Moser et al., 2000). These authors did observe improved litter gain and reduced backfat loss with increased Val, which could also be a function of Val:Leu increasing, with additional Val counteracting the negative impact of high Leu. In the current study, Leu:Lys ratio increased from 130% to 153%, and the Val:Leu ratio decreased from 65% to 55% as the dietary soybean meal concentration increased. Although feed-grade Val was added to the diet to maintain a similar Val:Lys ratio, Val may not have been adequate to counteract increasing Leu. Rather, it may be suggested that diets should also be balanced for Val:Leu to mitigate negative effects on feed intake from increasing Leu as soybean meal or other protein sources (dried distiller’s grains with solubles) are increased in lactation diets (Cemin et al., 2019).

Soybeans are known to contain several antinutritional factors, a few being trypsin inhibitors, lectin, raffinose, and stachyose (Rackis, 1975; Gu et al., 2010). While the heat applied during soybean meal processing typically inactivates the majority of trypsin inhibitors and lectin, raffinose and stachyose maintain their structure through processing (Zdunczyk et al., 2011). Monogastrics lack alpha-galactosidase, the enzyme necessary to break down raffinose and stachyose in the upper intestinal tract, resulting in fermentation in the lower gut. This can cause the production of short-chain fatty acids and gases, which leads to flatulence, diarrhea, and increased catabolism of dietary protein (Zdunczyk et al., 2011). While antinutritional factors were not measured in the soybean meal in the current study, it could be hypothesized that the decrease in feed intake in sows consuming diets containing 35% soybean meal may be due to increased concentrations of the antinutritional factors mentioned above. Interestingly, we also observed that the variation in ADFI within treatment was greater as the concentration of soybean meal increased. This might indicate that some sows can tolerate higher levels of soybean meal compared with others and this warrants further investigation.

Serum urea nitrogen measures the circulating nitrogen concentration, which is derived from both dietary nitrogen (from the metabolism of CP in the diet) and muscle catabolism. The present study observed an increase in serum urea nitrogen concentration with increasing soybean meal and concomitantly dietary CP. (Yang et al., 2000a) fed diets containing increased Lys and CP from soybean meal and also observed an increase in serum urea nitrogen. These authors also speculated that the increase in serum urea nitrogen could be a potential cause for decreased feed intake as they had observed in a previous study (Yang et al., 2000b). To differentiate the cause in increased serum urea nitrogen being derived from dietary CP or endogenous protein catabolism, creatinine was measured in the present study.

Circulating creatinine is used to indicate BP catabolism. Yang et al. (2009) observed increased creatinine at weaning when sows had been fed 1.0% vs. 1.3% total Lys during lactation, suggesting that protein mobilization was increased with the low Lys diet. Touchette et al. (1998) decreased sow BP mobilization by increasing SID Lys via increasing soybean meal. In the current study, an increase in sow BW, backfat, and maternal BL loss during lactation was observed with increasing soybean meal concentration, which demonstrates that the sow is mobilizing BL reserves. Thus, the observed increase in serum urea nitrogen with no change in creatinine is reflective of an increase in circulating nitrogen from dietary CP as soybean meal increased without a change in BP mobilization.

Increasing dietary soybean meal concentration resulted in no effect on the growth of suckling pigs even though greater changes in sow BW loss occurred with increasing dietary soybean meal concentration. Similarly, previous studies did not observe a difference in litter growth as soybean meal concentration increased from 19.3% to 34% (Gourley et al., 2017) or 24.6% to 34% (Greiner et al., 2018). This would suggest that modern sow genotypes will support high litter growth by mobilizing body reserves, even when feed intake is limited as demonstrated by the sows fed the high soybean meal diet in the current study. Additionally, this would suggest that the amount of Lys supplied in the current diets was adequate to meet the demand for litter growth with SID Lys intake ranging from 59.9 to 54.6 g/d for 25% to 35% soybean meal diets, respectively.

Despite the changes in BW and maternal BP during lactation, there was no evidence for a difference in WEI. When Lys is undersupplied during lactation, the subsequent reproductive performance can be negatively affected due to increased BP mobilization (Huang et al., 2013; Gourley et al., 2017). This effect is likely only observed after sows have lost greater than 12% of protein stores during lactation (Clowes et al., 2003). In the present study, sows were projected to only lose 2% maternal BP when fed 35% soybean meal diets. Thus, BP change was not great enough to elicit a negative impact in WEI, again suggesting that the range of 54.6–59.9 g/d SID Lys was adequate to support subsequent reproductive performance. Additional research is needed to determine if the magnitude of BP and BL loss occurring over multiple lactation periods from consuming high soybean meal concentration diets could lead to negative lifetime reproductive performance.

In summary, increasing soybean meal concentration from 25% to 35% decreased voluntary feed intake in lactating sows, with the greatest magnitude of change occurring as soybean meal was increased from 30% to 35%. Interestingly, there was no evidence for feed intake to be affected in the first 7 d after farrowing. This suggests that the decreased feed intake is not a result of the initial transition from a relatively low soybean meal level in the gestation diet compared with the lactation diet. There was no impact on litter growth or WEI; however, sows fed diets with 35% soybean meal had the greatest farrow-to-wean weight loss and backfat loss, which could affect future reproductive performance or longevity within the herd.

*Conflict of interest statement*. The authors declare no conflict of interest.
